# ﻿A new glassfrog species of the genus *Centrolene* (Amphibia, Anura, Centrolenidae) from Cordillera del Cóndor, southern Ecuador

**DOI:** 10.3897/zookeys.1149.96134

**Published:** 2023-02-22

**Authors:** Paul Székely, María Córdova-Díaz, Daniel Hualpa-Vega, Santiago Hualpa-Vega, Diana Székely

**Affiliations:** 1 Museo de Zoología, Universidad Técnica Particular de Loja, San Cayetano Alto, calle París s/n, 110107, Loja, Ecuador Universidad Técnica Particular de Loja Loja Ecuador; 2 Laboratorio de Ecología Tropical y Servicios Ecosistémicos (EcoSs-Lab), Facultad de Ciencias Exactas y Naturales, Departamento de Ciencias Biológicas y Agropecuarias, Universidad Técnica Particular de Loja, San Cayetano Alto s/n, 110107, Loja, Ecuador Ovidius University Constanţa Constanța Romania; 3 Research Center of the Department of Natural Sciences, Faculty of Natural and Agricultural Sciences, Ovidius University Constanţa, Al. Universității no.1, 900470, Constanța, Romania Fundación Green Jewel, Av. Pío Jaramillo y John Kennedy Loja Ecuador; 4 Fundación Green Jewel, Av. Pío Jaramillo y John Kennedy, Loja, Ecuador Ovidius University Constanţa Constanţa Romania

**Keywords:** Amphibians, DNA, phylogenetics, tadpoles, tropical Andes, vocalizations

## Abstract

Based on an integrative taxonomical approach, using molecular, morphological, and bioacoustics data, a new species of glassfrog of the genus *Centrolene* is described from Refugio de Vida Silvestre El Zarza, southern Ecuador. *Centrolenezarza***sp. nov.** is a medium sized species, easily distinguished from all other glassfrogs by its unique combination of characters, such as a shagreen dorsum with elevated warts corresponding to white spots, an evident tympanum, half or more than half of the upper parietal peritoneum covered by iridophores, iridophores absent on all visceral peritonea, including the pericardium, a lobed liver lacking iridophores, males with small projecting humeral spines, the outer edges of forearms and tarsus with a row of enameled warts that often continue into the external edges of Finger IV and/or Toe V, and white or yellowish white iris with thick black reticulations. The new species is closely related to a currently undescribed species and superficially resembles *C.condor*, *C.pipilata*, *C.solitaria*, *C.altitudinalis*, and *C.daidalea*. The tadpole and advertisement and courtship calls are described, and the threats to the species survival, mainly represented by habitat loss and contamination due to mining activities, are briefly discussed.

## Abstract (es)

Se describe una nueva especie de rana de cristal del género *Centrolene*, del Refugio de Vida Silvestre El Zarza, sur del Ecuador, basada en un enfoque de taxonomía integrativa, utilizando datos moleculares, morfológicos y bioacústicos. La especie *Centrolenezarza***sp. nov.** es de tamaño mediano, que se distingue fácilmente de todas las demás ranas de cristal por su combinación única de caracteres tales como dorso de piel rugosa con verrugas elevadas que corresponden a manchas blancas, tímpano evidente, la mitad o más del peritoneo parietal superior cubierto por iridóforos, iridóforos ausentes en todos los peritoneos viscerales, incluido el pericardio, hígado lobulado sin iridóforos, machos con pequeñas espinas humerales salientes, borde externo de los antebrazos y el tarso con hilera de verrugas esmaltadas que a menudo continúan en los bordes externos del Dedo IV y/o del Dedo V del pie, e iris blanco o blanco amarillento con reticulaciones negras gruesas. La nueva especie está estrechamente relacionada con una especie no descrita y se parece superficialmente a *C.condor*, *C.pipilata*, *C.solitaria*, *C.altitudinalis* o *C.daidalea*. Se describen renacuajos, cantos de aviso y cortejo, y se discuten brevemente las amenazas para su supervivencia, representadas por la pérdida de hábitat y la contaminación debido a las actividades mineras.

## ﻿Introduction

The charismatic glassfrogs belong to the Neotropical family Centrolenidae Taylor, 1951 that currently contains ca. 150 species classified into 12 genera (Guayasamin et al. 2009, 2020). These generally small, arboreal frogs share a unique morphology and behavior that makes them readily distinguishable: a green dorsum in most species, completely or partially translucent venter (hence the name of glassfrogs), humeral spines in males of some species, out-of-water deposition of eggs along streams, and forward-directed eyes (Guayasamin et al. 2020). Ecuador, despite its small size, has the second largest number of glassfrogs after Colombia, with 63 species from ten genera, 20 of them being endemic to the country (Guayasamin et al. 2020, 2022).

*Centrolene* Jiménez De La Espada, 1872 is the type genus for the family Centrolenidae, and is the third richest in number of species, after *Nymphargus* Cisneros-Heredia & McDiarmid, 2007 and *Hyalinobatrachium* Ruiz-Carranza & Lynch, 1991a. Currently there are 24 described species in the genus, and six more are listed as incertae sedis (Guayasamin et al. 2009, 2020). For most of the *Centrolene* species some DNA sequences are available (Guayasamin et al. 2008; Castroviejo-Fisher et al. 2014; Twomey et al. 2014), and these were included in the latest phylogenetic analysis of the genus presented by Guayasamin et al. (2020). For three species we still lack molecular data: *Centrolenepaezorum* Ruiz-Carranza, Hernández-Camacho & Ardila-Robayo, 1986 and *C.solitaria* (Ruiz-Carranza & Lynch, 1991c) from Colombia and *C.lemniscata* Duellman & Schulte, 1993 from Peru.

Currently, 12 *Centrolene* species have been reported from Ecuador, two being considered endemic (Guayasamin et al. 2020): *C.pipilata* (Lynch & Duellman, 1973) from the Amazonian slope of the Ecuadorian Andes, in the north and *C.condor* Cisneros-Heredia & Morales-Mite, 2008 from Cordillera del Cóndor, in the south. Additionally, four species are known from the Amazonian slopes of the Andes: *C.charapita* Twomey, Delia & Castroviejo-Fisher, 2014, *C.huilensis* Ruiz-Carranza & Lynch, 1995b, *C.medemi* (Cochran & Goin, 1970), and *C.sanchezi* Ruiz-Carranza & Lynch, 1991b. Herein we describe a new species of *Centrolene* from Cordillera del Cóndor, based on an integrative taxonomy approach, combining molecular, morphological and bioacoustics data.

## ﻿Materials and methods

### ﻿Specimen collection and study site

Field work was carried out between January 2020 and September 2022 in Refugio de Vida Silvestre El Zarza (Zamora Chinchipe province, southern Ecuador; 3.8341°S, 78.5458°W; datum WGS84; 1400–1680 m a.s.l.). Refugio de Vida Silvestre El Zarza (El Zarza wildlife refuge) is a national protected area founded in 2006 with the main aim of preserving some of Cordillera del Cóndor’s biological richness, with emphasis on amphibians and the Amazonian tapir. The refuge protects 3696.31 ha of evergreen lower montane forest and important water systems. Field work was carried out during the day and night (usually between 12h00–01h00), through intensive visual encounter surveys and auditory surveys. The distribution map was designed with QGis software and created using a digital elevation model obtained from JAXA/METI ALOS PALSAR Data (https://search.asf.alaska.edu/) and displayed via ASF DAAC.

All collected specimens were photographed alive, euthanized using 20% benzocaine, fixed in 10% formalin, and stored in 70% ethanol. Tissue samples for genetic analyses were preserved in 96% ethanol. Two egg clutches were collected and transported to the laboratory in order to raise and describe the tadpoles. Hatchlings and tadpoles were preserved in alcohol (as DNA samples) and 10% formalin (for the morphological analysis) in various developmental stages. Examined and referred specimens are housed at Museo de Zoología, Universidad Técnica Particular de Loja, Loja, Ecuador (**MUTPL**), Museo de Historia Natural Gustavo Orcés, Escuela Politécnica Nacional (**MEPN**), and Museo de Zoología, Pontificia Universidad Católica del Ecuador, Quito, Ecuador (**QCAZ**). Research permits were issued by the Ecuadorian Ministry of Environment (MAE-DNB-CM-2015-0016, MAAE-ARSFC-2020-0727, and MAATE-DBI-CM-2021-0181).

### ﻿Morphological analysis

For the description of qualitative and quantitative morphological characters, as well as the format of the description, we follow Cisneros-Heredia and McDiarmid (2007) and Guayasamin et al. (2020). Sex was determined by the presence of vocal slits, humeral spines and/or by gonadal inspection. Coloration of live specimens was based on field notes and digital photographs. All specimens were weighted (body mass: BM) before euthanasia using a My Weigh Triton T3 portable scale with 0.01 g precision. Measurements were taken under a stereo microscope, with a Vernier caliper, and rounded to the nearest 0.1 mm. Specimens were measured for the following morphometric variables:

**SVL** snout-vent length, distance from the tip of snout to posterior margin of vent;

**HW** head width, widest portion of the head, measured at level of jaw articulation;

**HL** head length, distance from the tip of snout to posterior angle of jaw articulation;

**IOD** interorbital distance, shortest distance between upper eyelids;

**IND** internarial distance, distance between the inner edges of the narial openings;

**EW** upper eyelid width, the perpendicular distance to the outer edge of the eyelid;

**ED** eye diameter, distance between anterior and posterior borders of eye;

**EN** eye-nostril distance, distance from posterior margin of nostril to anterior margin of eye;

**TD** tympanum diameter, horizontal distance between peripheral borders of tympanic annulus;

**FL** femur length, length of femur from vent to knee;

**TL** tibia length, length of flexed leg from knee to heel;

**FoL** foot length, distance from proximal margin of inner metatarsal tubercle to tip of Toe IV;

**HaL** hand length, distance from proximal edge of palmar tubercle to the tip of Finger III;

**3DW** width of disc on Finger III, greatest width of disc of Finger III.

Measurements are given as mean ± SD.

The developmental stages of embryos, hatchlings, and larvae were identified using the classification by Gosner (1960). Larval characters and description follow the terminology recommended by Mijares-Urrutia (1998), McDiarmid and Altig (1999), Anstis (2013), and Schulze et al. (2015). Photographs of tadpoles were taken of live specimens in a small glass tank and of the mouthparts on preserved specimens under a stereo microscope.

### ﻿Molecular analysis

Genomic extraction, amplification, and sequencing were as described in Székely et al. (2020) and the newly generated DNA sequences were deposited in GenBank (Appendix 1). For the phylogenetic analysis we used sequences of two mitochondrial ribosomal genes (*12S* and *16S* rRNA) and one nuclear gene (*POMC*) from 36 individuals of 28 species corresponding to 27 different localities from Colombia, Ecuador, Peru, and Venezuela (Appendix 1). We used all the GenBank-available sequences for *Centrolene* and ten new sequences (for two species) generated by our study. As outgroups, we used all the available sequences of *Nymphargus*, as well as sequences of *Chimerellamariaelenae* (Cisneros-Heredia & McDiarmid, 2006), *Espadaranacallistomma* (Guayasamin & Trueb, 2007), *Cochranellamache* Guayasamin & Bonaccorso, 2004, *Teratohylamidas* (Lynch & Duellman, 1973), *Sachatamiapunctulata* (Ruiz-Carranza & Lynch, 1995a), *Rulyranaflavopunctata* (Lynch & Duellman, 1973), *Vitreoranahelenae* (Ayarzagüena, 1992), *Celsiellavozmedianoi* (Ayarzagüena & Señaris, 1997), *Hyalinobatrachiumaureoguttatum* (Barrera-Rodriguez & Ruiz-Carranza, 1989), and *Ikakogitayrona* (Ruiz-Carranza & Lynch, 1991b). The tree was rooted with *Allophryneruthveni* Gaige, 1926.

The sequences were edited, assembled, and aligned (MAFFT algorithm with the G-INS-i iterative refinement method; Katoh and Standley 2013) using the program Geneious Prime (Biomatters Ltd.). The edited alignments of *12S*, *16S* and *POMC* sequences were visually inspected to correct alignment errors in PhyDE (Müller et al. 2010), concatenated into a single matrix, and then used for the phylogenetic analyses. The analyses were based on a 2457 bp dataset (961 bp for *12S*, 895 bp for *16S*, and 601 bp for *POMC*). The aligned and concatenated matrix is available at https://doi.org/10.5281/zenodo.7557286.

We follow the glassfrog taxonomy proposed by Guayasamin et al. (2009). Phylogenetic relationships were inferred using both Maximum Likelihood (ML) and Bayesian Inference (BI). We used PartitionFinder v. 2.1.1 (Lanfear et al. 2017) to select the best partition scheme with the corrected Akaike Information Criterion (AICc) as a model of selection. PartitionFinder identified three partition schemes (best model in parentheses): *12S* and *16S* (GTR+I+G), *POMC* 1^st^ position (TRN+G), and *POMC* 2^nd^ and 3^rd^ position (TRN+I+G). ML analyses were conducted in GARLI v. 2.1 (Zwickl 2006) performing 1000 tree searches (four independent searches, two with the “streefname” set to random and two set to stepwise, with 250 replicates each) and node support was assessed with 1000 bootstrap replicates. BI analysis was implemented in MrBayes 3.2.6 (Ronquist et al. 2012), the Markov chain Monte Carlo runs being performed twice, independently, for 70 million generations, with trees sampled every 1000 generations until convergence (*p* < 0.001) and consensus trees were summarized after discarding the initial 25% as burn-in. More details about how tree searches were performed are presented in Székely et al. (2020). The phylograms were edited with FigTree (Rambaut 2014).

A priori, we deemed that a tree node had “strong support” when its bootstrap value was > 75 and its Bayesian posterior probability was > 0.95, “moderate support” for 50–75 and 0.90–0.95, and “weak support” or non-resolved for values lower than 50 and 0.90, respectively (Vogel et al. 2020). Uncorrected genetic *p*-distances were calculated for *16S* with MEGA6 (Tamura et al. 2013) and are presented in Suppl. material 1.

### ﻿Bioacoustic analysis

We analyzed advertisement and courtship calls recorded in the field and in the laboratory. The calls were recorded in the field using an Olympus LS-11 Linear PCM Recorder and a RØDE NTG2 condenser shotgun microphone; in the laboratory we used a Tascam DR-100 MKIII Recorder with incorporated microphone. All recordings were made at 44.1 kHz sampling frequency and 16-bit resolution, in WAV file format. Air temperature and humidity were measured with a Lascar Electronics, model EL-USB-2-LCD data logger (accuracy: ± 0.5 °C; ± 5%). All analyzed call recordings are deposited in original form, full length at Fonoteca UTPL (record IDs are provided in Suppl. material 2). Acoustic analysis was conducted using Raven Pro 1.6 (K. Lisa Yang Center for Conservation Bioacoustics at the Cornell Lab of Ornithology). We measured the temporal parameters from the oscillograms and the spectral parameters from spectrograms obtained with the Hanning window function, DFT: 512 samples, 3 dB filter bandwidth: 124 Hz, and a 50% overlap (Székely et al. 2020).

The terminology and procedures for measuring call parameters follow Cocroft and Ryan (1995), Toledo et al. (2015) and Köhler et al. (2017), with a call-centered approach to distinguish between a call and a note (sensu Köhler et al. 2017). The following temporal and spectral parameters were measured and analyzed: (1) call duration: time from the beginning to the end of a call (for both single-note and multi-note calls); in the case of single-note calls this is the same as a note duration; (2) inter-call interval: the interval between two consecutive calls, measured from the end of one call to the beginning of the consecutive call (only for multi-note calls); (3) call rate: number of calls/minute, measured as the time between the beginning of the first call and the beginning of the last call (only for multi-note calls); (4) pulse duration: time measured from one amplitude minimum to the next amplitude minimum of a pulse; (5) pulse rate: number of pulses/second, measured as the time between the beginning of the first pulse and the beginning of the last pulse; (6) dominant frequency: the frequency containing the highest sound energy, measured along the entire call; and (7) the 90% bandwidth, reported as frequency 5% and frequency 95%, or the minimum and maximum frequencies, excluding the 5% below and above the total energy in the selected call (Székely et al. 2020).

## ﻿Results

### ﻿Phylogeny

**Figure 1.**
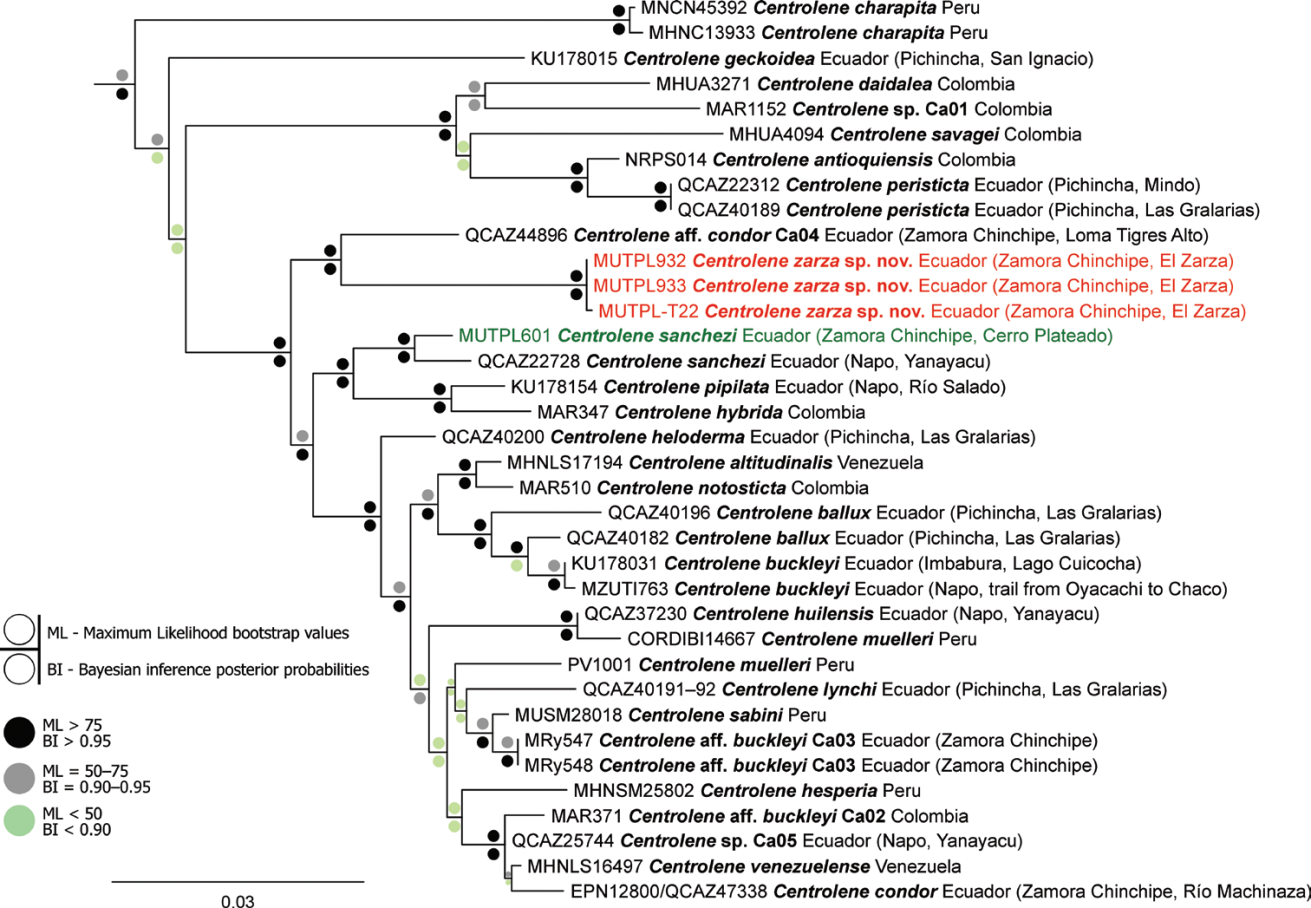
Maximum likelihood phylogram of *Centrolene*. The analysis is based on 2457 base pairs of concatenated mitochondrial DNA from *12S* and *16S*, and nuclear DNA from *POMC* gene fragments. Outgroup is not shown; the tree was routed with *Allophryneruthveni*. In red, the new species and in green a sequence newly generated by the present study. The catalog number, species name, country, and in the case of Ecuadorian species province and short locality names, are shown next to each terminal (associated data are listed in Appendix 1).

The Bayesian and Maximum likelihood phylogenetic trees showed very similar topologies, with minor differences in the position of some of the unresolved branches, mostly with stronger BI support (Fig. 1). We recovered *Centrolene* as monophyletic, with strong support in the BI (posterior probabilities = 0.99), but with only moderate support in the ML analysis (bootstrap values = 65.4). Overall, the phylogenetic tree of our analysis showed the same topology as the last one constructed for the genus by Guayasamin et al. (2020), but with differences in the position of *C.charapita*, *C.geckoidea* Jiménez de la Espada, 1872 and of several unresolved branches. These differences are most likely a consequence of the different gene sampling scheme, as we used only three genes for our analysis.

The new species is closely related to an undescribed species, the candidate species Ca04 from Guayasamin et al. (2020) identified in their tree as Centroleneaff.condor. These two are the sister group of a branch that includes 15 species and three candidate new species and are part of a strongly supported clade (bootstrap values = 94.7; posterior probabilities = 1) that contains almost 3/4 of all the *Centrolene* species (Fig. 1). Uncorrected *p*-genetic distances for the gene *16S* between the new species and Centroleneaff.condor range from 3.4% to 3.6% and the other members of the genus from 4.0% to 9.9% (Suppl. material 1).

### ﻿Taxonomy

**Class Amphibia Blainville, 1816**

**Order Anura Duméril, 1805**

**Superfamily Centrolenoidea Taylor, 1951**

**Family Centrolenidae Taylor, 1951**

**Subfamily Centroleninae Taylor, 1951**

#### Genus *Centrolene* Jiménez de la Espada, 1872

##### ﻿ Centrolene zarza sp. nov.

Taxon classificationAnimaliaAnuraCentrolenidae

33433A64-FE9F-5EF0-8677-DE06DBC4B54F

https://zoobank.org/BE900409-8243-4494-8B40-FE76C52BC571

Figs 2 , 3 , 4 , 5 , 6 , 7 , 8 , 9 Common English name: Zarza Glassfrog Common Spanish name: Rana de cristal del Zarza

###### Etymology.

The specific name *zarza* is a noun in apposition and refers to the species’ type locality: Refugio de Vida Silvestre El Zarza. This relatively small wildlife refuge conserves an impressive biodiversity with countless species of plants and birds, more than 50 species of amphibians and reptiles and several emblematic mammals, like the Amazonian tapir, jaguar, oncilla or the spectacled bear. It is surrounded by active mining concessions and thus fulfills an important role as a conservation island for the region, with an urgent need to expand connectivity between the reserve and neighboring conservation areas.

###### Type material.

**Figure 2.**
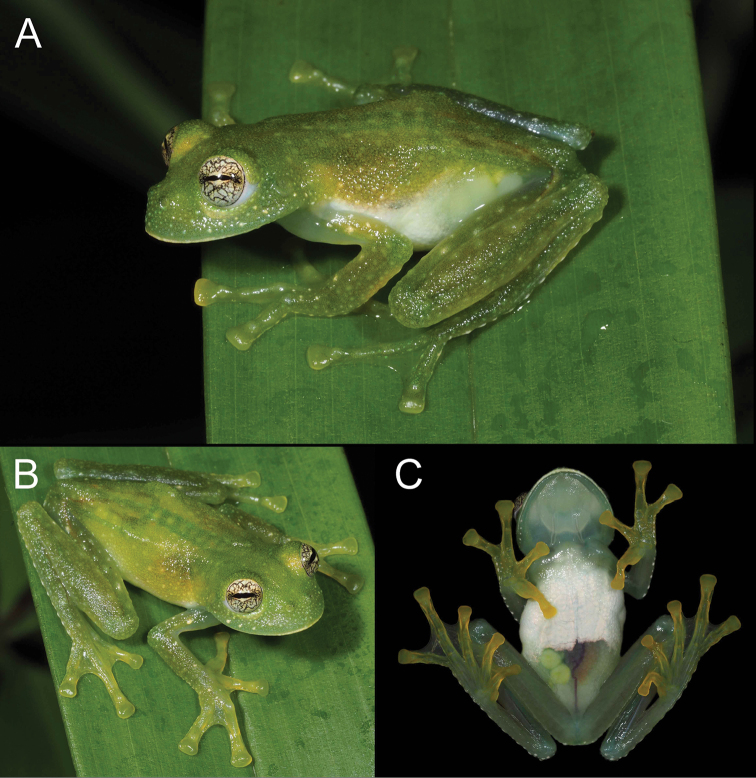
Holotype of *Centrolenezarza* sp. nov. (MUTPL 932, adult female), SVL 25.5 mm, in life **A** lateral view **B** dorsolateral view **C** ventral view.

**Figure 3.**
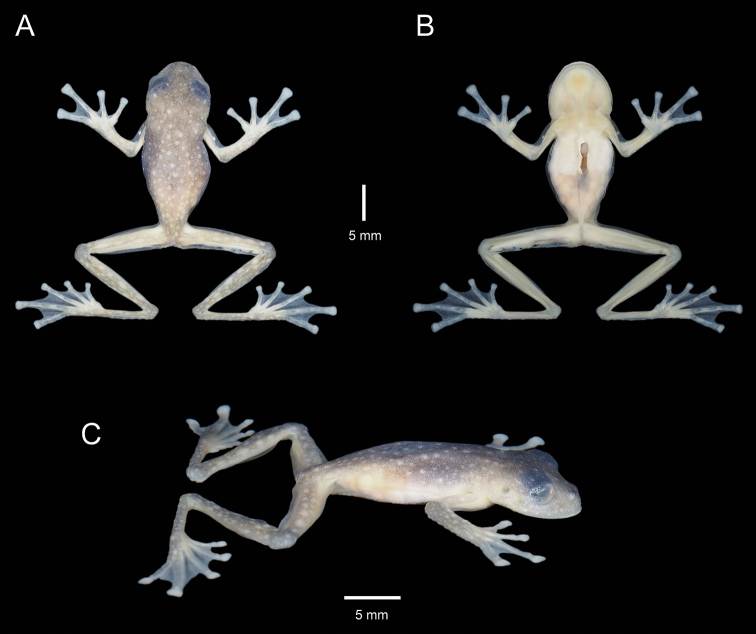
Holotype of *Centrolenezarza* sp. nov. (MUTPL 932, adult female) in preservative **A** dorsal view **B** ventral view **C** lateral view.

***Holotype*.**MUTPL 932 (field no. SC 425; Figs 2, 3, 5A), an adult female from Ecuador, Zamora Chinchipe Province, Refugio de Vida Silvestre El Zarza, quebrada “Las Mariposas” (3.8341°S, 78.5458°W; datum WGS84), 1434 m a.s.l., collected by Joselyn Vinueza, Santiago Hualpa-Vega, María Córdova-Díaz, Daniel Hualpa-Vega, Angel Hualpa, Dalton Morocho, Luis León, and Ramiro Sarango on 10 October 2020.

**Figure 4.**
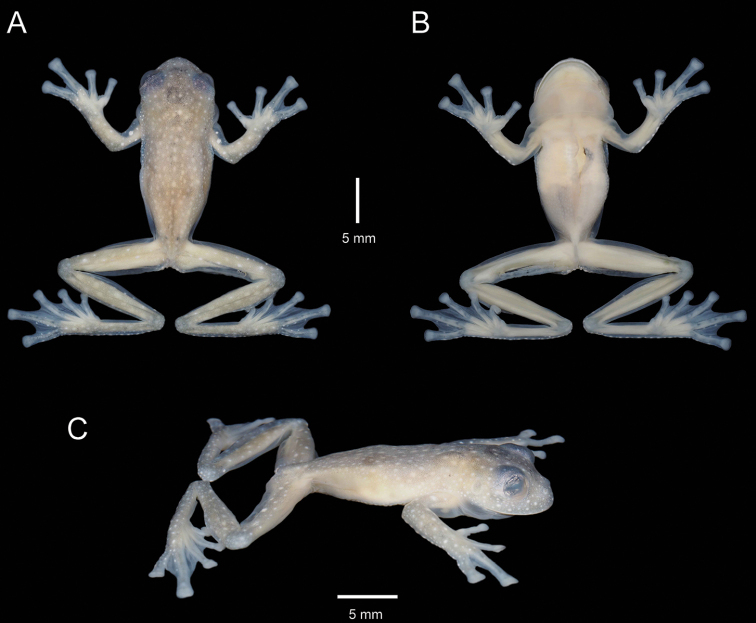
Paratype of *Centrolenezarza* sp. nov. (MUTPL 1023, adult male), SVL 23.2 mm, in preservative **A** dorsal view **B** ventral view **C** lateral view.

**Figure 5.**
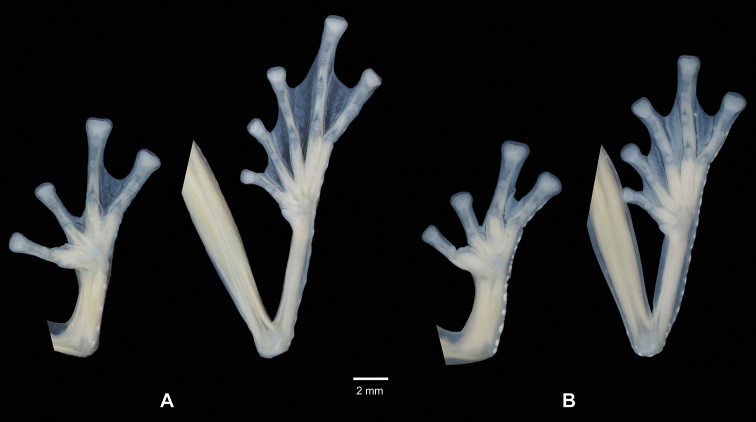
Palmar view of hand and plantar view of foot of **A** holotype of *Centrolenezarza* sp. nov. (MUTPL 932, adult female) and **B** paratype (MUTPL 1023, adult male) in preservative.

**Figure 6.**
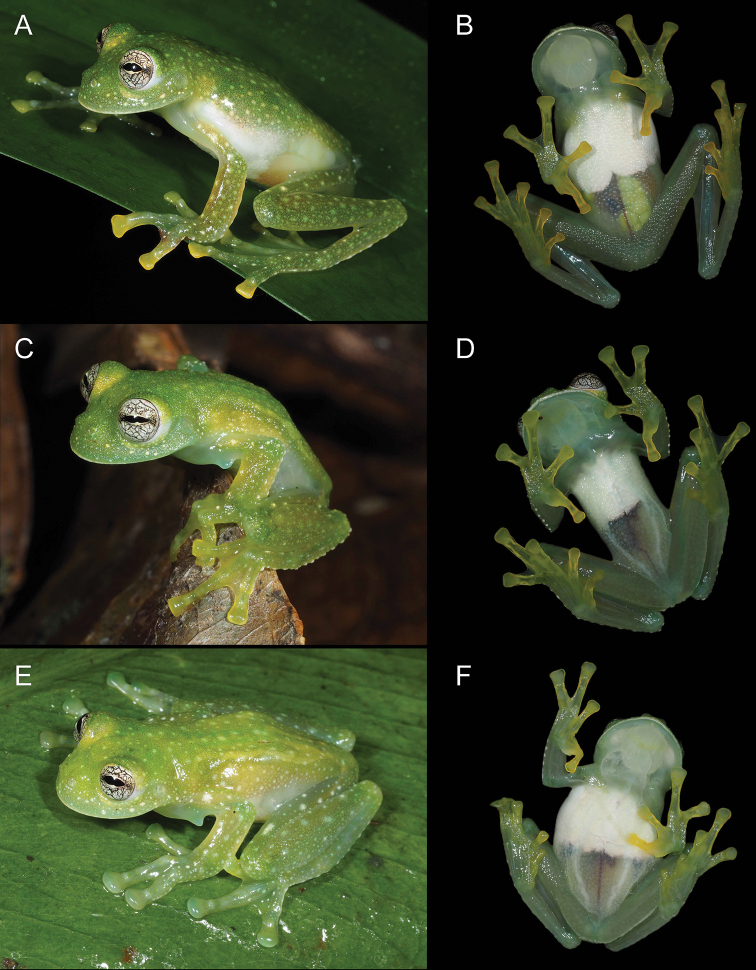
Morphological variation of *Centrolenezarza* sp. nov. in live specimens **A, B** female, paratype (MUTPL 1051) **C, D** male, paratype (MUTPL 933) **E, F** male, paratype (MUTPL 1022).

***Paratypes*.** (1 female, 5 males). MUTPL 933 (field no. SC 428; Fig. 6C, D) an adult male from Refugio de Vida Silvestre El Zarza, quebrada “Las Mariposas” (3.8371°S, 78.5424°W), 1461 m a.s.l. collected by Luis León, Dalton Morocho, Ramiro Sarango, Santiago Hualpa-Vega, María Córdova-Díaz, Daniel Hualpa-Vega, Angel Hualpa and Joselyn Vinueza on 10 October 2020; MUTPL 1022 (field no. SC 435; Fig. 6E, F), MUTPL 1023 (field no. SC 436; Figs 4, 5B), and MUTPL 1024 (field no. SC 437), adult males from Refugio de Vida Silvestre El Zarza, quebrada “Las Mariposas” (3.8376°S, 78.5421°W), 1469 m a.s.l., collected by Santiago Hualpa-Vega, María Córdova-Díaz, Daniel Hualpa-Vega, Camilo López, Dalton Morocho, Dalton Bustán and Luis León on 24 January 2021; MUTPL 1050 (field no. SC 443) adult male and MUTPL 1051 (field no. SC 444; Fig. 6A, B) adult female from Refugio de Vida Silvestre El Zarza, quebrada “Las Mariposas” (3.8373°S, 78.5423°W), 1471 m a.s.l., collected by Santiago Hualpa-Vega, María Córdova-Díaz, Daniel Hualpa-Vega, Joselyn Vinueza, Luis León, Dalton Morocho and Álex Armijos on 13 March 2021.

###### Diagnosis.

We assign this species to *Centrolene* based on phylogenetic evidence (Fig. 1) and on the general morphological similarity to other members of the genus (presence of humeral spines in males, liver divided into lobes and the hepatic peritoneum lacking an iridophore layer, and green bones in life). *Centrolenezarza* has the following combination of characters: (1) dentigerous processes of vomers ovoid, in transverse row between the choanae, separated medially by distance slightly lower than the width of processes; each process bearing 3–5 teeth; (2) snout rounded in dorsal view, sloping in profile; nostrils slightly elevated, producing depression in the internarial area; canthus rostralis not evident in dorsal view, rounded in cross section; (3) tympanic annulus and tympanic membrane evident but with coloration similar to that of surrounding skin; tympanum large, its diameter ~ 46% of eye diameter; weak supratympanic fold present, slightly concealing the upper margin of the tympanum; (4) dorsal skin shagreen with elevated, and some enameled, warts corresponding to white spots; (5) ventral skin coarsely areolate; ventral surfaces of thighs below vent with a pair of large, round, flat tubercles, flat tubercles (subcloacal warts); cloacal region bordered ventrally by many enameled, white, warts; (6) half or more than half of the upper parietal peritoneum covered by iridophores (condition P3); iridophores absent on all visceral peritonea, including the pericardium (condition V0); (7) liver lobed, lacking iridophores (condition H0); (8) adult males with small projecting humeral spines, round vocal slits and large subgular vocal sac; (9) webbing absent between Fingers I and II, basal between II and III, moderate between outer fingers: III2^+^–2IV; (10) webbing between toes moderate: I1-– -2II1-–2III1-–2IV2–1^+^V; (11) outer edge of forearms and tarsus with row of enameled warts that often continue into the external edges of Finger IV and/or Toe V; fingers and toes with broad lateral fringes; (12) unpigmented Type I nuptial pads present in males; concealed prepollex; (13) Finger I shorter than Finger II; (14) diameter of eye ~ 2× wider than disc on Finger III; (15) in life, dorsum light green with many white or whitish, elevated, spots and flecks of various sizes; bones green; (16) in preservative, dorsal surfaces greyish with white spots; (17) in life, iris white or yellowish white with thick and thin black reticulations; rounded points on the upper and lower side of the iris and no circumpupillary ring; (18) fingers and toes yellowish, usually lacking melanophores from the dorsal surfaces, except for Finger IV and Toes IV and V; (19) males call from the upper surfaces of leaves; advertisement call consisting of a high pitched, pulsed, single note, with every call/note featuring three clearly distinguishable pulses and a mean dominant frequency of 5309.8 Hz; courtship call composed by multi-noted, pulsed calls of usually five notes/call and a mean dominant frequency of 5127.4 Hz; (20) fighting behavior unknown; (21) egg clutches attached to the upper side of leaves; clutch size of 13–33 embryos (*n* = 2); probably without parental care; (22) tadpoles with elongated, oval-depressed body; sinistral spiracle; vent tube situated medially, caudal and with dextral opening; tail 2.4× the length of the body; labial tooth row formula 0/2(1) in Gosner 26 but without tooth rows in Gosner 31; mostly pinkish coloration; (23) medium body size (sensu Guayasamin et al. 2020), SVL 25.5–27.0 mm in adult females (*n* = 2) and 23.2–26.2 mm in adult males (24.1 ± 1.21, *n* = 5).

###### Comparisons with similar species.

Due to its unique combination of characters, *Centrolenezarza* is easily distinguished from all other glassfrog species. The few congener species that generally resemble *C.zarza*, specifically that have green dorsum with white spots and/or flecks, are as follows: *C.condor*, has a very different general habitus with a more robust body, smaller eyes, less evident tympanum and dark bluish-black/brown flecks and punctuations along with the white flecks (vs. slender body, larger eyes, evident tympanic annulus and tympanic membrane and only white spots and/or flecks in *C.zarza*); *C.pipilata*, has a dorsum with yellowish-white flecks and diffuse dark green/black marks, and a distinct prepollex (vs. only white spots and/or flecks and concealed prepollex); *C.sanchezi*, has a smaller body size and the presence of white warts in an area that extends from below the eye to the insertion of the arm (vs. absence of the white warts); specimens of the *C.buckleyi* species complex that have white spots have less evident markings, dentigerous processes of vomers without teeth, less evident tympanum, condition V1 of the visceral peritonea and also live at much higher elevations of 2050–3070 m (vs. dentigerous processes of vomers with teeth and condition V0 of the visceral peritonea).

Some congener species have a similar habitus, but live in other countries: *C.solitaria* (one of the few species of *Centrolene* for which we lack molecular data) is endemic to the Andes of Colombia and has green spots along with the white flecks, iridophores covering parts of the gastrointestinal peritoneum and the males lack humeral spines (vs. only white spots and/or flecks, iridophores absent on all visceral peritonea and males with humeral spines); *C.altitudinalis* (Rivero, 1968) is endemic to Andes of Mérida State from Venezuela (Barrio-Amorós et al. 2019), and has golden brown iris and lacks teeth on the dentigerous processes of vomers (vs. iris white or yellowish white with thick and thin black reticulations and dentigerous processes of vomers with teeth); *C.daidalea* (Ruiz-Carranza and Lynch 1991c), is reported from Colombia and Venezuela, and has black spots along with the white ones, iridophores covering parts of the gastrointestinal peritoneum and the males lack humeral spines (vs. only white spots, iridophores absent on all visceral peritonea and males with humeral spines).

Somewhat similar species of the genus which live on the Pacific versant of the Ecuadorian Andes are *C.ballux* (Duellman & Burrowes, 1989), that has a minute body size, a distinct prepollex and lacks teeth on the dentigerous processes of vomers (vs. medium body size, concealed prepollex and dentigerous processes of vomers with teeth), and *C.heloderma* (Duellman, 1981) which has a unique pustular dorsum, condition V1 of the visceral peritonea and vomers lacking teeth (vs. shagreen dorsum with elevated warts, condition V0 of the visceral peritonea and vomers with teeth). Finally, species from other genera that superficially resemble *C.zarza* are *Cochranellaresplendens* Lynch & Duellman, 1973, which has iridophores in pericardium and peritonea covering intestines and stomach and the males lack humeral spines (vs. iridophores absent on all visceral peritonea and males with humeral spines) and *Nymphargusposadae* (Ruiz-Carranza & Lynch, 1995a) that has almost indistinguishable tympanum, condition P2 and V1, and the males lack humeral spines (vs. evident tympanum, condition P3 and V0, and males with humeral spines).

###### Description of the holotype.

Adult female (MUTPL 932; Figs 2, 3, 5A), medium sized, with many large and small yellowish white eggs. Head slightly narrower than the body, wider than long, head length 91% of head width, head width 34% of SVL, head length 31% of SVL; snout relatively short, snout to eye distance 13% of SVL, eye-nostril distance 24% of head length; snout rounded in dorsal view, sloping in profile; nostrils slightly elevated producing a shallow depression in the internarial area; canthus rostralis indistinct in dorsal view, rounded in cross section; loreal region slightly concave; lips non-flared; moderate sized eyes (sensu Guayasamin et al. 2020), eye diameter 11% of SVL, 34% of head length; eye-nostril distance 69% of eye diameter; eye diameter 93% of IOD; eyes directed anterolaterally at ~ 40° from midline, slightly visible from below; upper eyelid width 85% of IOD; tympanic annulus and tympanic membrane evident, but the membrane with coloration similar to that of surrounding skin; tympanum oriented slightly dorsolaterally; tympanum large (sensu Guayasamin et al. 2020), its diameter ~ 46% of eye diameter; weak supratympanic fold present, slightly concealing the upper margin of the tympanum; choanae large, ovoid, not concealed by palatal shelf of maxillary arch, closer to the distal margin of the dentigerous processes of vomers than to the margin of mouth; dentigerous processes of vomers ovoid, in transverse row between the choanae, separated medially by distance slightly lower than the width of processes; each process bearing three teeth; tongue just slightly longer as wide, not notched posteriorly, and only ~ 1/5 of posterior part not adherent to the floor of mouth.

Skin of dorsal surfaces shagreen with elevated, and some enameled, warts corresponding to white spots; throat smooth; ventral skin coarsely areolate; ventral surfaces of thighs below vent with a pair of large, round, flat tubercles (subcloacal warts); cloacal opening directed posteriorly at upper level of thighs, no distinct cloacal sheath; cloacal region bordered ventrally by many enameled (white) warts.

Upper arm thin, forearm somewhat robust; outer edge of forearms with row of enameled warts that continue into the external edges of Finger IV; hand length 34% of SVL; palmar tubercle large, elliptical; thenar tubercle large, ovoid; subarticular tubercles prominent, round and round in section; numerous round palmar supernumerary tubercles present, much smaller than subarticular tubercles; relative lengths of fingers I < II < IV < III; concealed prepollex; fingers with broad lateral fringes; webbing absent between Fingers I and II, basal between II and III, moderate between outer fingers: III2^+^–2IV (Fig. 5A); bulla absent; discs on fingers expanded, truncate; disc pads nearly triangular.

Hindlimbs long, slender; femur length 55% of SVL; tibia length 57% of SVL; foot length 48% of SVL; outer edge of tarsus with row of enameled warts that often continue into the external edges of Toe V; inner edge of tarsus bearing a long fold; inner metatarsal tubercle large, elliptical; outer metatarsal tubercle indistinct; subarticular tubercles rounded and flat; plantar supernumerary tubercles inconspicuous; relative length of toes I <II < III < V < IV; toes with broad lateral fringes; webbing between toes moderate: I1-–-2II1-–2III1-–2IV2–1^+^V (Fig. 5A); discs on toes expanded, truncate, lacking papillae; disc pads nearly triangular.

###### Coloration of holotype.

In life (Fig. 2): dorsum light green, head and dorsal surfaces of arms and of hindlimbs darker green, with many white or whitish, elevated, spots and flecks of various sizes. The green vertebral column, sacrum, ileum, and urostyle visible through the skin (Fig. 2B). Flanks white or transparent (Fig. 2A); venter with more than half of the upper parietal peritoneum covered by white iridophores; lower parietal peritoneum transparent with part of the large intestine, urinary bladder, and some large yellowish white eggs visible (Fig. 2C); ventral vein red (Fig. 2C). Fingers and toes yellowish (more evident ventrally; Fig. 2C) lacking melanophores from the dorsal surfaces, except for Fingers III and IV and Toes IV and V. Iris yellowish white with thick and thin black reticulations and no circumpupillary ring. Bones green.

In preservative (Fig. 3): dorsal surfaces greyish with white spots; vertebral column, sacrum, ileum and urostyle no longer visible through the skin (Fig. 3A). Throat yellowish, upper parietal peritoneum on the venter white, lower part transparent (Fig. 3B). Melanophores absent from hands and feet, except few present on dorsal surfaces of Finger III and many on Finger IV and Toes IV and V.

###### Measurements of holotype

**(in mm).**SVL 25.5; HW 8.7; HL 7.9; IOD 2.9; IND 2.1; EW 2.5; ED 2.7; EN 1.9; snout to eye distance 3.4; TD 1.4; FL 14.1; TL 14.5; FoL 12.2; HaL 8.6; 3DW 1.6.

###### Body mass of holotype.

1.05 g.

###### Variation.

Morphometric variation is shown in Table 1. The females are larger, with slender bodies and longer limbs (Figs 3, 6A, B). The males have more robust bodies, slightly thicker forearms and have humeral spines (Figs 4, 6C–F). One male (MUTPL 1022, Fig. 6E) had a slightly little lighter dorsal coloration, but overall, no significant variation in dorsal coloration, iris coloration or pattern of the spots or flecks was observed between the encountered individuals. The green bones of limbs and vertebral column were visible dorsally, through the skin, in all specimens.

**Table 1.** Morphometric characters of *Centrolenezarza* sp. nov. Body mass (in grams), measurements (in mm) and morphological proportions (in percentages) of adult females and males; values are given as mean ± SD (range). Female body mass includes eggs.

Character	Females (*n* = 2)	Males (*n* = 5)
Body mass (BM)	1.21 (1.05–1.37)	0.89 ± 0.08 (0.84–1.02)
Snout-vent length (SVL)	26.3 (25.5–27.0)	24.1 ± 1.21 (23.2–26.2)
Head width (HW)	9.1 (8.7–9.5)	8.4 ± 0.34 (7.9–8.7)
Head length (HL)	8.1 (7.9–8.4)	6.9 ± 0.43 (6.6–7.6)
Interorbital distance (IOD)	2.8 (2.8–2.9)	2.8 ± 0.13 (2.6–2.9)
Internarial distance (IND)	2.2 (2.1–2.2)	2.0 ± 0.09 (1.9–2.1)
Upper eyelid width (EW)	2.3 (2.1–2.5)	2.2 ± 0.24 (1.9–2.6)
Eye diameter (ED)	2.8 (2.7–3.0)	2.7 ± 0.18 (2.5–2.9)
Eye-nostril distance (EN)	2.0 (1.9–2.2)	1.8 ± 0.22 (1.6–2.1)
Tympanum diameter (TD)	1.3 (1.3–1.4)	1.2 ± 0.10 (1.2–1.4)
Femur length (FL)	14.8 (14.1–15.6)	13.2 ± 0.54 (12.7–14.0)
Tibia length (TL)	15.4 (14.5–16.2)	13.9 ± 0.49 (13.2–14.6)
Foot length (FoL)	12.5 (12.2–12.8)	11.5 ± 0.77 (10.6–12.7)
Hand length (HaL)	9.0 (8.6–9.5)	8.3 ± 0.31 (8.0–8.8)
Width of disc on Finger III (3DW)	1.6 (1.6–1.7)	1.4 ± 0.20 (1.1–1.6)
HW/SVL	33.9–35.0	32.6–36.9
HL/SVL	30.8–30.9	26.2–32.8
HL/HW	88.4–90.8	77.2–88.9
EN/HW	21.4–22.8	18.1–25.0
EN/HL	23.6–25.7	20.4–31.1
EN/IOD	63.8–78.2	55.4–71.9
ED/HW	31.2	31.6–33.3
ED/HL	34.4–35.3	37.5–43.2
ED/IOD	93.1–107.3	91.2–107.5
EN/ED	68.5–72.9	54.5–78.8
TD/ED	45.8–46.3	40.4–50.9
3DW/ED	57.4–57.6	44.0–58.2
EW/IOD	74.5–84.5	66.7–96.2
IND/IOD	72.4–80.0	64.9–76.9
IOD/HW	29.1–33.5	31.0–34.8
IOD/HL	32.9–36.9	36.8–43.2
FL/SVL	55.1–57.8	53.5–58.2
TL/SVL	56.9–60.0	55.6–60.6
FoL/SVL	47.4–47.8	45.7–50.6
HaL/SVL	33.7–35.0	33.0–35.8

###### Eggs and tadpoles.

**Figure 7.**
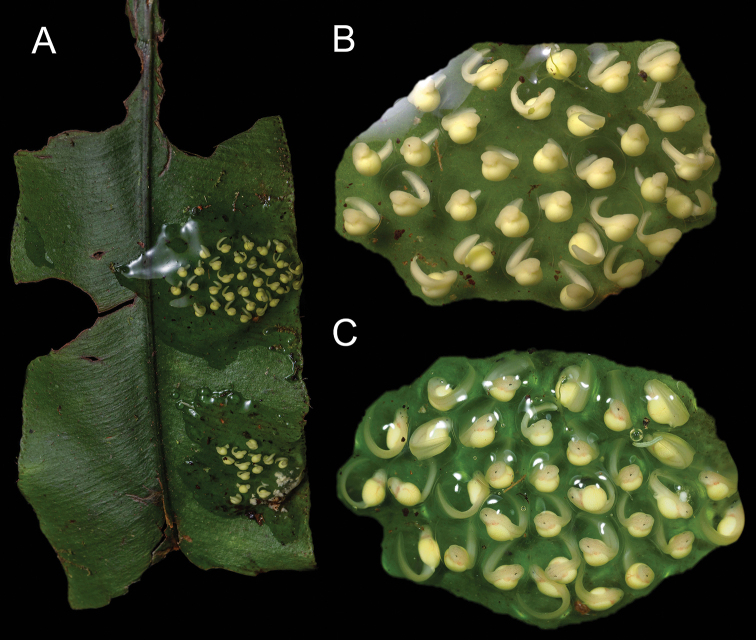
Egg-clutches of *Centrolenezarza* sp. nov. collected from the type locality (MUTPL-T22) **A** the egg-clutches attached to a leaf (10 June 2021) **B** hatchlings (sensu McDiarmid and Altig 1999) in stage Gosner 20 (10 June 2021) **C** hatchlings in stage Gosner 22 (13 June 2021).

Two egg clutches in stage Gosner 19 (Fig. 7) were collected from the type locality (3.8379°S, 78.5418°W; 1460 m a.s.l.) on 9 June 2021 (MUTPL-T22). Both egg clutches were attached to the upper side of a leaf at ~ 3 m above the stream. The clutches contained 13 and 33 embryos; no adults were observed guarding the eggs or in the near proximity. The tadpoles hatched in the laboratory after 5 days and survived for more than 6 months, until 2 January 2022. They developed well in the beginning, but halted their development at Gosner stage 31 (ca. 20 November), and unfortunately started to die in January 2022 without completing their metamorphosis. It is possible that the tadpoles died due to inadequate rearing conditions, or their death was produced by chytridiomycosis (see the discussion section).

**Figure 8.**
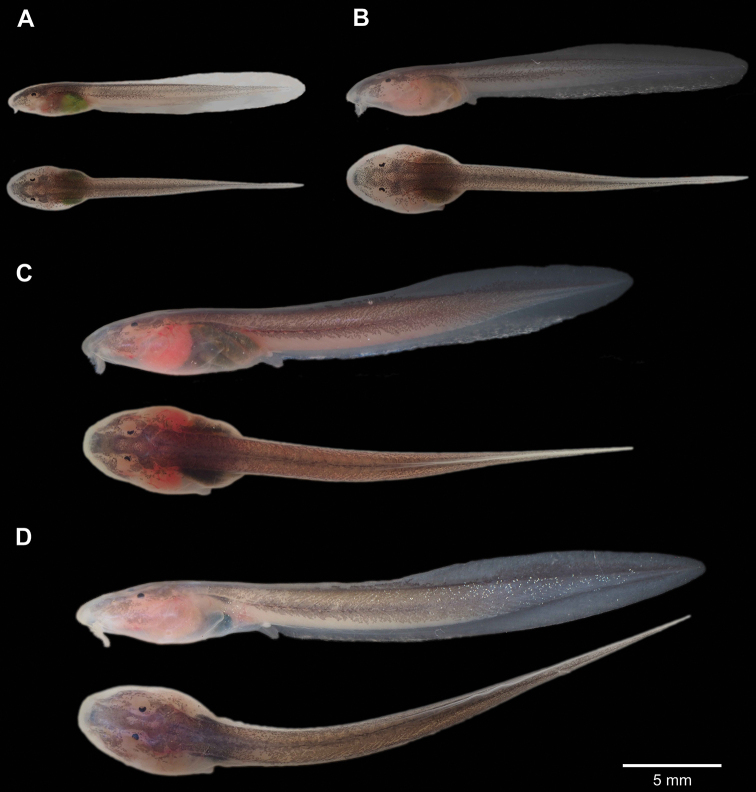
Hatchlings (sensu McDiarmid and Altig 1999) and tadpoles of *Centrolenezarza* sp. nov. (MUTPL-T22) **A** Gosner stage 24 (26 June 2021) **B** Gosner stage 25 (5 August 2021) **C** Gosner stage 26 (23 August 2021) **D** Gosner stage 31 (20 November 2021).

The tadpoles of *C.zarza* (Fig. 8) belong to Type IV tadpole of Orton (1953), and the exotroph, lotic, and burrower ecomorphological guild of McDiarmid and Altig (1999). The following description is based on tadpoles at Gosner stages 26 and 31 (from the MUTPL-T22 series). For the tadpoles in Gosner stage 31 the total length was 26.9–34.7 mm (31.9 ± 2.67, *n* = 6) and the body length was 8.2–10.4 mm (9.5 ± 0.73, *n* = 6), body length being ~ 30% of total length.

Body elongated, oval-depressed, wider than high; snout rounded in dorsal view and sloped and rounded in lateral view. Eyes located on dorsal surface of head and C-shaped (at least until Gosner stage 31). Nostrils positioned dorsally, protruding, with very small narial apertures oriented anteriorly. Spiracle short, single, sinistral, located at the posterolateral region of the body; spiracular opening slightly below body axis, oriented posteriorly and upwards (dorsoposterior orientation). Vent tube situated medially, short, attached to the ventral fin (caudal), with a dextral opening directed postero-ventrally. Tail long, ~ 2.4× the length of the body, with subacute tip. Dorsal fin originating at ca. mid-length of tail; myotomes of tail musculature weakly visible in the first half of tail length.

**Figure 9.**
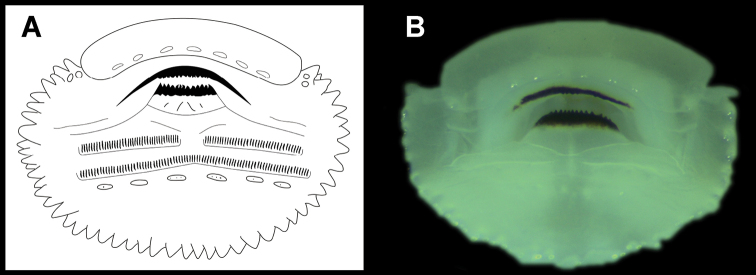
Oral apparatus of the tadpoles of *Centrolenezarza* sp. nov. (MUTPL-T22) **A** Gosner stage 26 **B** Gosner stage 31.

Oral disc large (oral disc width ~ 65% of body width), not emarginated, located near tip of snout, directed anteroventrally, protruding ventrally but not laterally (Fig. 8), beyond body. Marginal papillae uniserial, large, distributed around oral disc (~ 43–47 papillae); large part of the anterior (upper) margin of labium lacking papillae and instead having an involuted fold with smooth surface, but with a row of submarginal flattened papillae-like ridge in the proximity of the upper jaw sheath (Fig. 9). Upper jaw sheath broadly arched, slender (with less than half depth of upper jaw cartilages keratinized) and with serrated edge; lower jaw sheath slightly U-shaped, slender (with less than half depth of lower jaw cartilages keratinized) and with serrated edge (Fig. 9). Labial tooth row formula (LTRF) 0/2(1), P-1 with medial gap, and with a row of submarginal flattened papillae-like ridge (composed by seven or eight papillae) on the posterior (lower) labium in Gosner stage 26 (Fig. 9A). However, in Gosner stage 31, all the tooth rows were lost and only the tooth (dermal) ridges were visible beside the submarginal ridge of papillae (Fig. 9B). It is not clear if the loss of the tooth rows is a natural process throughout the metamorphosis or it is caused by disease or other factors (see the discussion section).

General coloration varied in the different developmental stages (Fig. 8). Hatchlings (sensu McDiarmid and Altig 1999) in Gosner stage 24 were slightly pinkish but with a special green coloration of the abdomen (Fig. 8A). The tadpoles had a more evident pinkish coloration in Gosner stage 25 (Fig. 8B) and became almost red by the Gosner stage 26 (Fig. 8C). However, after a couple of months, by the Gosner stage 31, they had lost the reddish coloration and had the body just slightly pink and the tail almost brown (Fig. 8D). We do not know if this discoloration was a natural developmental process or the tadpoles were actually suffering from a disease or had other problems.

###### Vocalizations.

**Figure 10.**
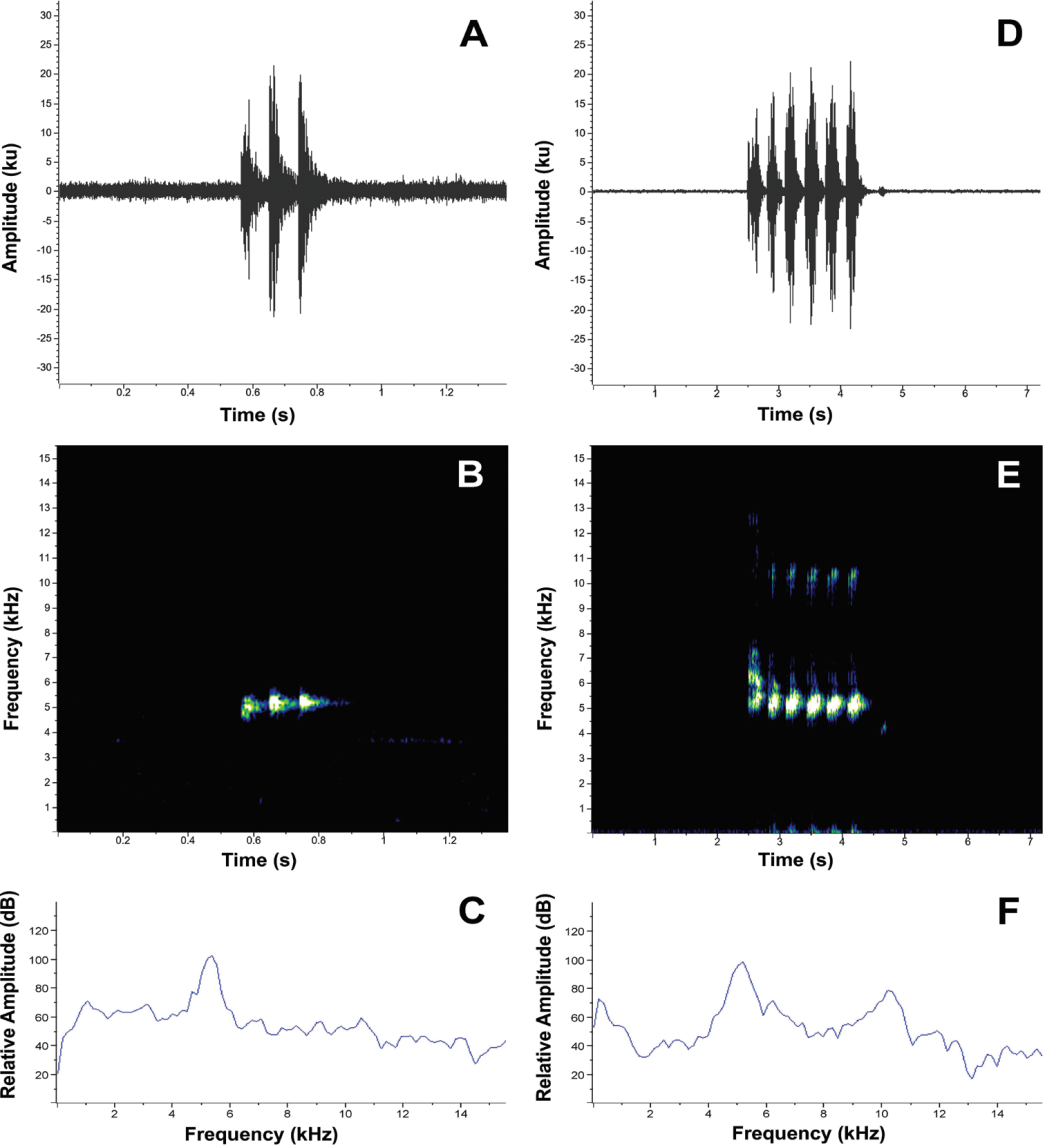
Vocalizations in *Centrolenezarza* sp. nov. Visual representation of the advertisement call (FUTPL-A 263; **A–C**) and courtship call (paratype MUTPL 933, FUTPL-A 261; **D–F**) **A** oscilogram of a single-noted call with the 3 pulses **B** spectrogram of a single-noted call **C** power spectrum of a single-noted call **D** oscilogram of a multi-noted call with 6 notes **E** spectrogram of a multi-noted call **F** power spectrum of a multi-noted call.

On 3 September 2022 we recorded the calls of several males from the type locality (Refugio de Vida Silvestre El Zarza, quebrada “Las Mariposas”, Suppl. material 2). The males were calling (advertisement calls sensu Wells 2007) from above, at several meters high, in the vegetation bordering a small stream. For the description of these calls we used two recordings: FUTPL-A 263 and FUTPL-A 264 (the detailed information of each of the separate recordings is presented in the Suppl. material 2). Because the males were calling out of reach, up in the trees, we were not able to distinguish and pinpoint the calling males and so the recordings contain the calls of several males. Thus, the call description is based on the calls of probably three of the closest calling males. For this reason, we were not able to measure some of the temporal parameters, like the inter-call interval and call rate. We used for the analysis only the calls clearly distinguishable in each of the recordings, which were not overlapped by other calls. The advertisement call of *C.zarza* is characterized by a high pitched, pulsed, single note, with every call/note featuring 3 clearly distinguishable pulses (Fig. 10A–C). The calls had a duration of 0.242–0.318 s (0.268 ± 0.02, *n* = 17), with a pulse duration of 0.072–0.159 s (0.089 ± 0.02, *n* = 51), and an average pulse rate of 11.8 pulses/s. The mean dominant frequency of the calls was 5309.8 Hz, with a mean 90% bandwidth of 5041.3–5487.2 Hz (Suppl. material 2). The fundamental frequency is not recognizable, and no harmonics are visible.

The call of paratype MUTPL 933 (FUTPL-A 261 and FUTPL-A 262) was recorded on 14 October 2020, on the first night that the specimen arrived in the laboratory. The animal was encountered in the type locality on a leaf at a height of 1.5 m near the stream while vocalizing. In laboratory, a recorder was left running all night, in order to record the call. The male was left in the same plastic bag in which it arrived from the field and had in its proximity, in a different bag, a female (the holotype). The male vocalized almost all night, but the call had a different structure from the typical advertisement call heard and recorded in the field (Fig. 10D–F). We identified this call as a courtship call (sensu Wells 2007), being composed not only by the single-noted calls, but mainly by multi-noted calls (usually of five notes/call, but up to six notes/call). The notes had the same structure as the single-noted calls, with the typical three pulses. Unfortunately, the pulses are not sufficiently visible in oscillograms or spectrograms in order to allow accurate measurements (Fig. 10D), probably due to the special conditions from the laboratory (echo from the walls, animal in plastic bag, etc.). The multi-noted calls had a duration of 0.581 to 1.905 s (1.300 ± 0.45, *n* = 18), depending on the number of notes/calls. The individual notes had a duration similar to the ones from the single-noted calls of the advertisement calls, just slightly longer: 0.244–0.378 s (0.304 ± 0.03, *n* = 80). The inter-call interval varied from 40.2 to 596.9 s (255.5 ± 171.23, *n* = 18) and the call rate was ~ 0.23 calls/min, or rather, ~ 14 calls/hour (Suppl. material 2). The frequencies were slightly lower than the ones from the advertisement calls, with the mean dominant frequency of 5127.4 Hz, and a mean 90% bandwidth of 4914.7–5411.2 Hz (Suppl. material 2). The fundamental frequency was not recognizable, but 2 to 3 harmonics were visible, although these could be artificially produced by the echo from the walls in the laboratory (Fig. 10E).

It seems that in this species the males emit multi-note courtship calls when they detect the nearby presence of females and are used to interact with them, as in our case, where the male was probably aware of the female’s presence. Similar behavior was observed in other glassfrog species (Greer and Wells 1980; Hutter et al. 2013) and it is well documented in various anuran species (see Wells 2007 for a detailed discussion). No multi-note calls were heard or recorded in the field but we did not witness any female-male interactions.

###### Distribution.

**Figure 11.**
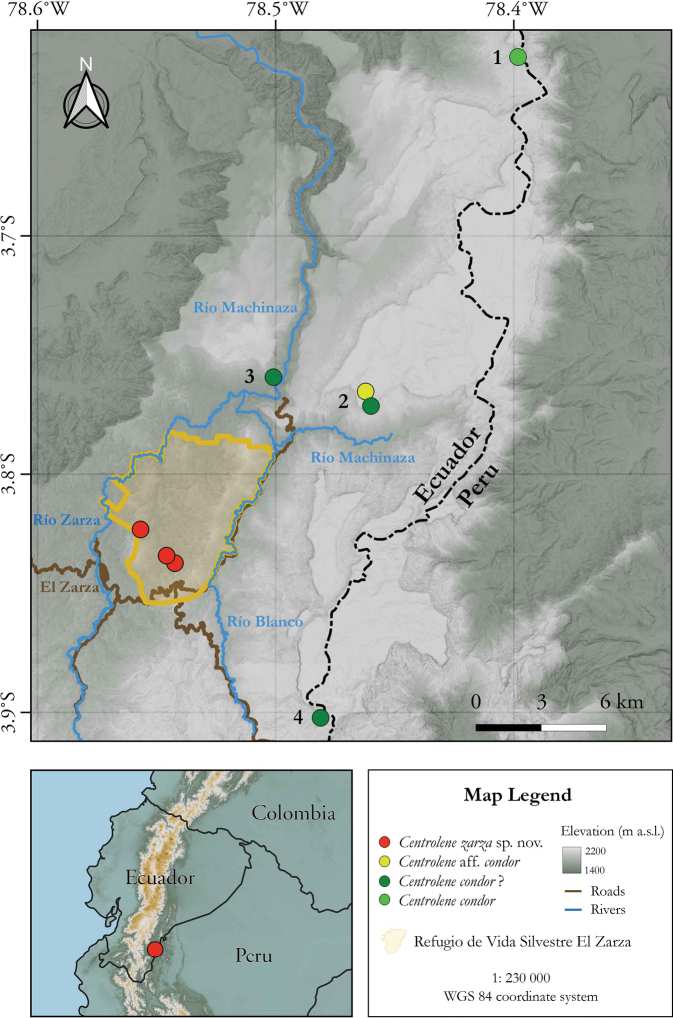
Distribution of *Centrolenezarza* sp. nov. Records are based on specimens deposited at the Museo de Zoología, Universidad Técnica Particular de Loja (MUTPL), Museo de Historia Natural Gustavo Orcés, Escuela Politécnica Nacional (MEPN) and Museo de Zoología, Pontificia Universidad Católica del Ecuador (QCAZ). 1. Destacamento Militar Cóndor Mirador, the type locality of *Centrolenecondor*. 2. Alto Machinaza, with the two collecting sites, Loma Tigres Alto (yellow dot) and Loma Tigres Bajo (green dot). 3. Río Machinaza – Sector Colibrí. 4. Paquisha Alto.

*Centrolenezarza* is currently known only from Refugio de Vida Silvestre El Zarza, Zamora Chinchipe province, southern Ecuador (Fig. 11). The specimens were encountered at an altitudinal range between 1434 and 1480 m a.s.l. in an evergreen lower montane forest ecosystem.

###### Natural history.

**Figure 12.**
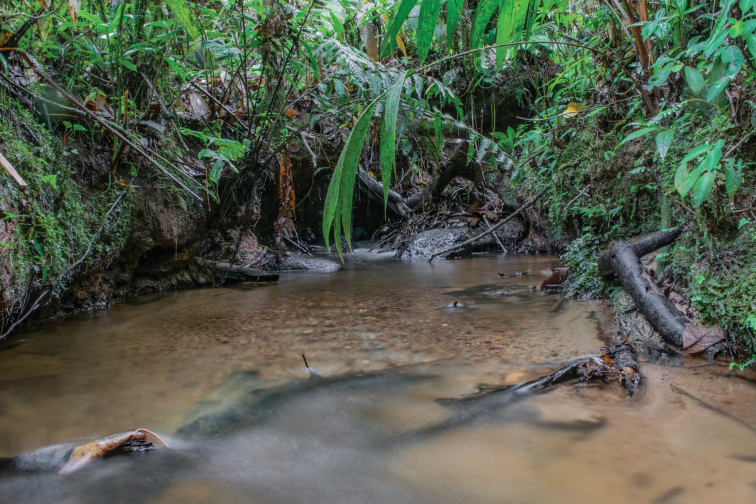
Habitat of *Centrolenezarza* sp. nov. in the type locality. Refugio de Vida Silvestre El Zarza, quebrada “Las Mariposas”.

This is a (locally) common species in the sense that the species presence was detected (seen or heard), in the proper habitat, in large or moderate numbers, on more than 50% of the sampling days/nights (Székely et al. 2020). All specimens were encountered during the night, on the upper surfaces of leaves of the vegetation bordering two small streams (Fig. 12). Calling males were heard during January, March, June, October, and November, but intense activity (many males calling from lower heights, 50 cm to 3 m above the streams) was recorded between October and March. The female paratype MUTPL 1051 was encountered nearby the calling male MUTPL 1050 at ~ 4 m high over the stream. However, no multi-note courtship calls were heard, nor was any direct interaction observed between these individuals. Observed syntopic glassfrog species were mainly *Espadaranaaudax* (Lynch & Duellman, 1973), in large numbers, but also *Nymphargusposadae*, *Chimerellamariaelenae*, and *Rulyranamcdiarmidi* (Cisneros-Heredia, Venegas, Rada & Schulte, 2008).

###### Conservation status.

*Centrolenezarza* is known, for now, from only two small streams inside the wildlife refuge, in an estimated area of less than 7 km^2^. Although this is a locally common species, which lives inside a nationally protected area, we recommend that *C.zarza* be categorized as Critically Endangered following the B1ab(i,ii,iii)+2ab(i,ii,iii) IUCN criteria (IUCN 2001) because: (1) its Extent of occurrence (EOO) and Area of occupancy (AOO) are estimated to be less than 7 km^2^; (2) it is known from only one locality; and (3) its habitats could be affected in the near future by mining activities (both legal or illegal), as the wildlife refuge is surrounded by active mining concessions.

## ﻿Discussion

### ﻿The *Centrolenecondor* “problem”

The Condor Glassfrog (*C.condor*) was described by Cisneros-Heredia and Morales-Mite in 2008 with one male specimen collected in 2003 and without molecular data (Cisneros-Heredia and Morales-Mite 2008). Unfortunately, since then no additional specimens were encountered or collected from the type locality, Destacamento Militar Cóndor Mirador in the Zamora Chinchipe province (Fig. 11). Thus, the (molecular) identity of this species, and the relationships with its congeners are still unknown. Between March 2008 and July 2012 Ana Almendáriz and her team from Museo de Historia Natural Gustavo Orcés, Escuela Politécnica Nacional (MEPN), conducted several expeditions (as part of the socio-environmental studies of the area needed for a mining company) in the southern sector of the Cordillera del Cóndor, specifically in an area known as Alto Machinaza (Fig. 11; Almendáriz et al. 2014). From Alto Machinaza and nearby areas (such Río Machinaza – Sector Colibrí and Paquisha Alto, Fig. 11) they collected several specimens identified as *C.condor*. With the data collected in these expeditions Ana Almendáriz and Diego Batallas expanded the distribution range of the species with more than 30 km to the south, provided data on its habitat and tadpoles and described the call (Almendáriz and Batallas 2012). These animals are morphologically very similar to *C.condor*, but without molecular confirmation we cannot be sure they are the same species, especially since the same authors noted some small morphological differences compared with the original description. One of these specimens (EPN 12800/QCAZ 47338; the specimen is maintained in the MEPN collection) was sequenced by researchers from QCAZ and it is currently labeled as *C.condor* in the phylogenetical tree of Guayasamin et al. (2020) and our own study (Fig. 1). Another specimen collected from Loma Tigres Alto (Alto Machinaza; Fig. 11) as a tadpole, was sequenced and identified as Centroleneaff.condor (QCAZ 44896, the candidate species Ca04) in Guayasamin et al. (2020). However, this specimen, which is the sister species of *C.zarza*, is positioned in a different branch of the tree, far from the “*C.condor*” collected from almost the same location (Río Machinaza – Sector Colibrí).

The fact that *C.zarza* is morphologically different from *C.condor* (see comparisons with similar species section) suggests that the current position of *C.condor* in the phylogenetic tree may be correct and that the specimens from Alto Machinaza and Paquisha Alto are indeed *C.condor* (or at least very similar, closely related species). For this reason, it is imperative that new material is collected from the type locality in order to clarify the exact position of *C.condor*. Unfortunately, this could be a difficult task to accomplish, as the whole area is already a mining company´s concession and access is restricted. Another option would be to obtain sequences from the formalized holotype using alternative extracting methods from degraded DNA (e.g., Bernstein and Ruane 2022).

### ﻿Conservation

The main threats for *C.zarza* are habitat loss and contamination due to mining activities, both legal and illegal. The whole southern sector of the Cordillera del Cóndor is threatened by imminent human colonization and settlement, agriculture and cattle raising, as well as gold and copper mining. The situation of Refugio de Vida Silvestre El Zarza, in this context, is of particular concern. For now, the wildlife refuge acts like a conservation island, being surrounded by large mining concessions and with active mining activities close to its northern borders. To make things worse, in recent years, signs of illegal mining activities were recorded inside the refuge. These activities are conducted especially in the streams of the reserve, and could affect, particularly but not exclusively, the survival of the glassfrogs that live in the wildlife refuge, especially due to water contamination. Currently, there are 6 species and one potentially new species of glassfrogs recorded from the refuge, although still others might remain to be discovered in the future.

Another threat for the survival of *C.zarza* could be chytridiomycosis, the infectious fungal disease caused by *Batrachochytriumdendrobatidis* (*Bd*), that has been linked to worldwide amphibian population declines (Berger et al. 1998; Lips et al. 2006; Skerratt et al. 2007). We did not carry out a survey to detect the pathogen presence in the amphibian populations from the refuge, but the death of our tadpoles raised in the laboratory could be attributed to the infection. Several studies have established a relationship between *Bd* infection and the occurrence of anomalies in the oral apparatus of tadpoles in various amphibian species (Fellers et al. 2001; Drake et al. 2007; Vieira et al. 2013) as chytridiomycosis affects only the keratinized tissues, which are restricted to the oral region (jaw sheaths and teeth) of tadpoles (Marantelli et al. 2004). In our case, tadpoles in Gosner stage 26 had two rows of tooth but by Gosner stage 31, all the tooth rows were lost and only the dermal ridges were visible (Fig. 9). Rueda-Almonacid (1994) recorded something similar in the case of *C.geckoidea*, where the tadpoles in Gosner 22 (one month after hatching) had two incomplete tooth rows on the anterior labium and three tooth rows on the posterior labium but, three months after hatching, all the tooth rows were lost.

However, it is possible that the loss of tooth rows is a natural (developmental) process. This could be particularly true in our case, as we did not observe oral deformities in the form of dekeratinization (depigmentation) of mouthparts (which are the typical symptoms of *Bd* infection; Navarro-Lozano et al. 2018), just the loss of the tooth rows. Also, it is possible that the loss of tooth was caused by other factors, as there are studies that have indicated that oral deformities are not always related to *Bd* infection (Rachowicz 2002; Blaustein et al. 2005; Navarro-Lozano et al. 2018) and could be attributed to other factors, like low temperatures (Rachowicz 2002), water contamination (Rowe et al. 1998) or diet (McDiarmid and Altig 1999). In order to resolve this issue, we plan to implement a survey in the near future to confirm or not the pathogen’s presence in the reserve and particularly in the threatened amphibian populations.

## Supplementary Material

XML Treatment for Centrolene zarza

## ﻿Appendix I

**Table A1.** Voucher, GenBank accession numbers and locality for the *Centrolene* species used in the phylogenetic analysis. With bold letters are marked the sequences generated by the present study. Ca01–Ca05 represent candidate new species from Guayasamin et al. (2020).

Species	Voucher number	GenBank accession no.			Locality
		*12S*	*16S*	*POMC*	
*Centrolenealtitudinalis*	MHNLS 17194	EU663333	EU662974	EU663165	Venezuela: Mérida, Quebrada Albarregas
*Centroleneantioquiensis*	NRPS 014	EU663336	EU662977	EU663167	Colombia: Antioquia, Vereda El Roble
*Centroleneballux*	QCAZ 40182	–	JX126954	–	Ecuador: Pichincha, Reserva Las Gralarias, road Calacalí – La Independencia
*Centroleneballux*	QCAZ 40196	KF639754	–	–	Ecuador: Pichincha, Reserva Las Gralarias, road Calacalí – La Independencia
*Centrolenebuckleyi*	KU 178031	EU663338	EU662979	EU663169	Ecuador: Imbabura, near Lago Cuicocha
*Centrolenebuckleyi*	MZUTI 763	MH844843	MH844849	–	Ecuador: Napo, trail from Oyacachi to Chaco
Centroleneaff.buckleyi Ca02	MAR 371	EU663339	EU662980	EU663170	Colombia: Cundinamarca, Parque Nacional Chingaza
Centroleneaff.buckleyi Ca03	MRy 547	MH844838	MH844844	–	Ecuador: Zamora Chinchipe
Centroleneaff.buckleyi Ca03	MRy 548	MH844839	MH844845	–	Ecuador: Zamora Chinchipe
*Centrolenecharapita*	MHNC 13933	KM068248	KM068256	–	Peru: Amazonas, La Oliva
*Centrolenecharapita*	MNCN 45392	KF639760	KF534358	–	Peru: Amazonas, La Oliva
*Centrolenecondor*	EPN 12800/ QCAZ 47338	–	MT225186	–	Ecuador: Zamora Chinchipe, Río Machinaza– Sector Colibrí
Centroleneaff.condor Ca04	QCAZ 44896	KF639755	JX126955	–	Ecuador: Zamora Chinchipe, Machinaza – Loma Tigres Alto
*Centrolenedaidalea*	MHUA 3271	EU663366	EU663007	EU663192	Colombia: Cesar, Vereda San Cayetano
*Centrolenegeckoidea*	KU 178015	EU663341	EU662982	–	Ecuador: Pichincha, 1 km SW San Ignacio
*Centroleneheloderma*	QCAZ 40200	KF639757	JX126956	–	Ecuador: Pichincha, Reserva Las Gralarias, Río Santa Rosa
*Centrolenehesperia*	MHNSM 25802	EU663345	EU662986	KF639777	Peru: Cajamarca, Quebrada Chorro Blanco
*Centrolenehuilensis*	QCAZ 37230	–	JX126959	–	Ecuador: Napo, Yanayacu Biological Station
*Centrolenehybrida*	MAR 347	EU663346	EU662987	EU663175	Colombia: Boyacá, Reserva Natural El Secreto
*Centrolenelynchi*	QCAZ 40191–92	KF639758	JX126957	–	Ecuador: Pichincha, Reserva Las Gralarias, road Calacalí – La Independencia
*Centrolenemuelleri*	CORDIBI 14667	–	KM068267	–	Peru: Amazonas, Puente – Vilcaniza
*Centrolenemuelleri*	PV 1001	KF639759	JX126958	KF639778	Peru: Amazonas, Chachapoyas, Cataratas de Gokta
*Centrolenenotosticta*	MAR 510	EU663351	EU662992	EU663180	Colombia: Norte de Santander, Vereda Piritama, Quebrada Piritama
*Centroleneperisticta*	QCAZ 22312	EU663352	EU662993	EU663181	Ecuador: Pichincha, Mindo Biology Station
*Centroleneperisticta*	QCAZ 40189	MT225171	–	–	Ecuador: Pichincha: Reserva Las Gralarias
*Centrolenepipilata*	KU 178154	EU663353	EU662994	KF639779	Ecuador: Napo, Río Salado, 1 km upstream from Río Coca
*Centrolenesabini*	MUSM 28018	–	JX126960	–	Peru: Cusco, Parque Nacional Manu nearby Pilco Grande
*Centrolenesanchezi*	MUTPL 601	**OP751416**	**OP751399**	**OP753140**	Ecuador: Zamora Chinchipe, Reserva Biológica Cerro Plateado
*Centrolenesanchezi*	QCAZ 22728	EU663337	EU662978	EU663168	Ecuador: Napo, Yanayacu Biological Station
*Centrolenesavagei*	MHUA 4094	EU663380	EU663020	EU663205	Colombia: Antioquia, Vereda El Retiro
*Centrolenevenezuelense*	MHNLS 16497	EU663360	EU663001	EU663186	Venezuela: Mérida, Cordillera de Mérida
*Centrolenezarza*	MUTPL 932	**OP751417**	**OP751400**	**OP753141**	Ecuador: Zamora Chinchipe, Refugio de Vida Silvestre El Zarza
*Centrolenezarza*	MUTPL 933	**OP751418**	**OP751401**	**OP753142**	Ecuador: Zamora Chinchipe, Refugio de Vida Silvestre El Zarza
*Centrolenezarza*	MUTPL-T22	–	**OP751402**	–	Ecuador: Zamora Chinchipe, Refugio de Vida Silvestre El Zarza
*Centrolene* sp. Ca01	MAR 1152	KM068295	KM068295	–	Colombia: Chocó, Corregimiento de Balboa
*Centrolene* sp. Ca05	QCAZ 25744	MT225170	–	–	Ecuador: Napo, Yanayacu Biological Station

## Supplementary materials

Supplementary material 1 Uncorrected genetic *p*-distances (%), for the mitochondrial gene *16S* for the *Centrolene* species Data type xlsx file https://binary.pensoft.net/file/814304This dataset is made available under the Open Database License (http://opendatacommons.org/licenses/odbl/1.0/). The Open Database License (ODbL) is a license agreement intended to allow users to freely share, modify, and use this Dataset while maintaining this same freedom for others, provided that the original source and author(s) are credited.Paul Székely, María Córdova-Díaz, Daniel Hualpa-Vega, Santiago Hualpa-Vega, Diana Székely

Supplementary material 2 Information regarding the *Centrolenezarza* sp. nov. call recordings and the bioacoustic measurements Data type xlsx file Explanation note Values are given as average ± SD (range) and *n* = sample size. https://binary.pensoft.net/file/814305This dataset is made available under the Open Database License (http://opendatacommons.org/licenses/odbl/1.0/). The Open Database License (ODbL) is a license agreement intended to allow users to freely share, modify, and use this Dataset while maintaining this same freedom for others, provided that the original source and author(s) are credited.Paul Székely, María Córdova-Díaz, Daniel Hualpa-Vega, Santiago Hualpa-Vega, Diana Székely
